# Effects of young age at presentation on survival in breast cancer

**DOI:** 10.1186/1471-2407-6-194

**Published:** 2006-07-20

**Authors:** Nagi S El Saghir, Muhieddine Seoud, Mazen K Khalil, Maya Charafeddine, Ziad K Salem, Fady B Geara, Ali I Shamseddine

**Affiliations:** 1 Department of Internal Medicine, American University of Beirut Medical Center, Beirut, Lebanon; 2 Department of Obstetrics & Gynecology, American University of Beirut Medical Center, Beirut, Lebanon; 3 Radiation Oncology of the American University of Beirut Medical Center, Beirut, Lebanon

## Abstract

**Background:**

Young age remains a controversial issue as a prognostic factor in breast cancer. Debate includes patients from different parts of the world. Almost 50% of patients with breast cancer seen at the American University of Beirut Medical Center (AUBMC) are below age 50.

**Methods:**

We reviewed 1320 patients seen at AUBMC between 1990 and 2001. We divided them in three age groups: Below 35, 35–50, and above 50. Data and survival were analyzed using Chi-square, Cox regression analysis, and Kaplan Meier.

**Results:**

Mean age at presentation was 50.8 years. 107 patients were below age 35, 526 between 35–50 and 687 patients above age 50. Disease stages were as follows: stage I: 14.4%, stage II: 59.9%, stage III: 20% and stage IV: 5.7%. Hormone receptors were positive in 71.8% of patients below 35, in 67.6% of patients 35–50 and in 78.3% of patients above 50. Grade of tumor was higher as age at presentation was lower. More young patients received anthracycline-based adjuvant chemotherapy. Of hormone receptor-positive patients, 83.8% of those below age 35 years, 87.76% of those aged 35–50 years, and 91.2% of those aged above 50 years received adjuvant tamoxifen. The mean follow up time was 3.7 +/- 2.9 years. Time to death was the only variable analyzed for survival analysis. Excluding stage IV patients, tumor size, lymph node, tumor grade and negative hormone receptors were inversely proportional to survival. Higher percentage of young patients at presentation developed metastasis (32.4% of patients below 35, as compared to 22.9% of patients 35–50 and 22.8% of patients above 50) and had a worse survival. Young age had a negative impact on survival of patients with positive axillary lymph nodes, and survival of patients with positive hormonal receptors, but not on survival of patients with negative lymph nodes, or patients with negative hormonal receptors.

**Conclusion:**

Young age at presentation conferred a worse prognosis in spite of a higher than expected positive hormone receptor status, more anthracycline-based adjuvant chemotherapy and equivalent adjuvant tamoxifen hormonal therapy in younger patients. This negative impact on survival was seen in patients with positive lymph nodes and those with positive hormonal receptors.

## Background

Breast cancer is the most common female cancer worldwide and is the second leading cause of cancer mortality among women in the United States [1]. In Lebanon, breast cancer is the most frequent malignancy in women and constitutes about one third of all female cancers [2,3]. While the median age at presentation is around 63 years in the United States and Western Europe, the median age at presentation in Lebanon is 48–52 years, similar to Mexico, Saudi Arabia and the palestinians; with an ASR of 46.7 per 100.000 in 1998 [3-8].

Many studies, from many parts of the world, have looked at the prognostic value of age at diagnosis in patients with breast cancer [9-19]. In 1994, Swanson, et al, after controlling for race, stage and treatment, found that mortality due to breast cancer is greatest in younger women[9]. Another study of invasive non-metastatic breast cancer in premenopausal women reported in 1993, young age had an adverse effect on cancer specific survival and relapse-free interval[10]. Most of these studies are hospital based and single institution studies. A recent population-based study from Switzerland was reported to show that young women present with larger tumors and more aggressive tumors but found no effect of age on survival[11]. Studies from Saudi Arabia and Singapore reported no adverse effect of young age on survival[12,13]. Factors considered to play a role in poor prognosis include more aggressive tumors, higher grades, and negative hormone receptors status. Recent studies have indicated a higher local relapse rate in young women with breast cancer treated with breast-conserving therapy [14-16] which emphasizes the role of type of treatment received as another factor to be studied when evaluating the effects of age on prognosis.

We aimed to assess the prognostic value of young age on the outcome of breast cancer, and to shed more light on the influence of clinical and pathological characteristics and treatment modalities on age and prognosis.

## Methods

### Study cohort

We reviewed the medical records of all 1320 breast cancer patients seen at the American University of Beirut Medical Center between 1990 and 2001. Study was approved by the Institutional Review Board (IRB) of the American University of Beirut Medical Center. Information was collected from the Hospital Inpatient Medical Records Department, Outpatient Departments, and Radiation Oncology Department. Additional follow-up information was collected by phone calls made to patients and/or their families, where necessary. The details of these interviews were recorded and the date of interview was used in our Cox and Kaplan Meier analyses. Fifty-seven patients were removed from the study because of missing information. And the remaining 1263 patients diagnosed between 1990 and 2001 were analyzed.

The date of last visit was used as the end date in Cox and Kaplan Meier analyses. In case the patient had passed away, the respondent or member of the family was asked to provide us with the date of death.

### Study variables

Variables were collected using a questionnaire which included: Sociodemographic data, past medical history and risk factors, pathologic characteristics and treatment details. Sociodemographic data collected included the age, residence, marital status. Past medical history included coronary artery disease, hypertension, diabetes and other malignancies. Risk factors included were age at menarche and menopause, family history of breast cancer, parity, living children, age at first conception, exposure to hormonal therapy as oral contraceptive pills (OCP) in premenopausal women or hormone replacement therapy (HRT) in postmenopausal women, and benign or low risk breast lesions. Pathologic data included information on tumor histology, size, grade, receptor status and axillary lymph nodes. Hormonal receptors were determined by immunohistochemistry and were considered positive when either the ER, PgR or both were positive. Hormone receptors were considered negative when concentration was below 10%.

Treatment data included type and duration of neo-adjuvant therapy, breast-conserving surgery or mastectomy, adjuvant chemotherapy, radiation therapy and hormonal therapy with tamoxifen which is given after chemotherapy. Follow-up data included information about local and distant sites of recurrences. Dates and causes of death of deceased patients were recorded.

### Statistical methods

The patients were divided into three age groups: patients less than 35  years of age (Group 1), patients between 35 and 49 years of age (Group 2),  and patients above 50 years of age (Group 3).

The data was analyzed using SPSS v.13.0 and STATA 7.0. Frequencies of all the variables in question were calculated. Univariate analysis includes frequencies, and bivariate analysis includes crosstabs (chi square) and t-test. In the multivariate analysis, Cox regression was used to estimate predictors of shorter survival using the backward method for insignificant variables elimination with a removal criteria at less than 0.1. Variables that are considered in this regression were age, presence of positive lymph nodes (LNs), grade and size of tumor, hormonal status, adjuvant chemoradiation and hormonal therapy.

Kaplan Meier curves were used to estimate time-to-death; in this case the log-rank was used to compare different groups. P < 0.05 was considered significant in all analysis.

## Results

Mean age at presentation was 50.8 ± 12.2 years. 56% of the patients were postmenopausal. The distribution of patients by age categories was as follows: 107 patients (8.1%) were less than 35 years of age, 526 patients (39.9%) were between 35 and 49 and 687 patients (52%) were aged 50 years and above.

Patients came from all over the country. 49.3 % (507 patients) were residents of the capital city Beirut. 7.5% from North Lebanon, 14.5% from South Lebanon, 5.9% from Bekaa region, and 20.5% from Mount Lebanon region. This distribution is similar to the population distribution in the country. Only 2.2% of the patients came from outside Lebanon.

238 patients (18.3%) had a family history of breast cancer. 87.5% were married with children. 12.5% never married and never had any children.

197 patients (15.4%) had a history of hypertension and 91 patients (7.1%) had diabetes. 342 patients (34%) had a history of smoking.

Stage distribution of the tumors was as follows: Stage I: 14.4%, stage II: 59.9%, stage III: 20.0%, and stage IV: 5.7%.

The median follow up time was 2.9 years.

## Clinical characteristics and treatment details

The clinical and pathological characteristics, status, and adjuvant chemotherapy of all patients grouped as <35 years (group 1), between 35–50 years (group 2) and >50 years (group 3) are summarized in table 1.

The three age-groups considered were comparable as far as the distribution of stage, tumor size and lymph node positive status Past medical history (including diabetes, CAD, and HTN) and family history of breast cancer were also similar across the three age groups mentioned.

68.6% of the young group (Group 1) had positive lymph nodes, as compared to 66.7% and 61.6% for groups 2 and 3 respectively. This difference was not significant (P value: 0.131).

On the other hand, hormonal receptors, grade and adjuvant chemotherapy were statistically different across the mentioned age groups. Table 1 shows that 78.5% patients who are older than 50 have positive hormonal receptors, followed by 71.6% of those who are less than 35 years of age, and finally 67.6% of the intermediate group.

There was a significant difference in the grade between the three groups. The grade was worse as the age at presentation decreased (P = 0.019). In fact, Grade III tumours constituted 49%, 42.1% and 35.8% of cases for patients <35, 35–50 and >50 years of age respectively.

Adjuvant chemotherapy was given to 76.5% and 72.8% of group 1 and group 2 respectively, while only to 53.1% of the older age group (P < 0.001). As for the type of chemotherapy, more anthracycline-based regimens were given to patients who were between 35 and 50 years old in 68.3% of the cases, and to only 56% in group 1 patients and 50.6% in group 3 patients (P < 0.001). This suggested that young patients were treated more aggressively than their older counterparts.

Adjuvant hormonal therapy was given to patients with positive hormone receptor in the three age groups were as follows: 62 patients of 74 (83.8%) aged less than 35 received adjuvant tamoxifen, 287 patients of 327 (87.76%) ages: 35–50 received tamoxifen, and 455 patients of 499 (91.2%) aged above 50 years received adjuvant tamoxifen. It was equivalent in the three age groups (P = 0.081: not significant). Patients reported in this series were seen before the results of adjuvant trials with aromatase inhibitors were published and all patients were treated with adjuvant tamoxifen. The survival in the young age group of women was worse in spite of the hormonal treatment

Higher proportion of patients in the youngest group develops metastasis after diagnosis; 32.4% compared to 22.9% and 22.8% for groups 1 and 2 respectively. However, p value of 0.098 suggests an insignificant difference in these proportions.

### Effects of various prognostic factors on survival in non-metastatic patients

Table 2 shows the results of the Cox regression analysis. This analysis was done to assess the variables that contribute most to the duration of survival. Patients who had Stage IV at presentation were removed from this table.

#### 1- Effect of tumor grade

Tumor grade is a significant prognostic factor for survival; grade II and grade III patients are 1.813 and 2.161 times more likely to have a shorter survival than grade I patients. P value was most significant for Grade III patients.

#### 2- Effect of positive hormonal receptors

Table 2 shows that patients with negative hormonal receptors are 65% less likely to survive than the group with positive hormonal receptors. As mentioned earlier, The distribution of adjuvant hormonal treatment in hormone receptor positive patients in the three age groups. was equivalent (P = 0.081). The survival in the young age group of women was worse in spite of the hormonal treatment

#### 3- Effect of tumor size

Tumor size played a major role in predicting survival. While those who have T_2 _lesions (2–5 cm) have 2.003 higher chance or shorter survival, patients with T_3 _lesions (>5 cm) reach a remarkable 3.099 higher risk of death than T_1 _(<2 cm) lesions patient with a 95% CI that reaches 5.89 times the risk of a T_1 _patient.

#### 4- Effect of positive lymph nodes

The presence of positive LNs had a more adverse effect on survival for the youngest group than for the other two groups. Lymph node positivity was inversely proportional to survival in our population (p < 0.001). In fact those who have lymph node invasion are 2.370 times more likely to die, controlling for the rest of the variables.

#### 5- Effect of age

**Figure 1:**
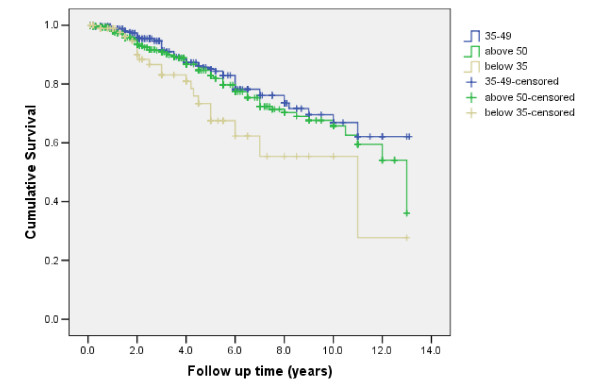
Stratified analysis of survival of breast cancer between three age groups (below 35, 35-50, and above 50): Patients with primary breast cancer below the age of 35 had a worse survival than the group 35-50 and group above age 50. The p-value of 0.03 shows that young age at presentation disadvantages survival.

The best survival was noted for the middle group that includes patients 35–49 years, followed by those who are >50 years, and the worst survival was found in the youngest group (Figure 1). When we stratified patients by age groups: Group 1 (<35), Group 2 (35–50) and Group 3 (>50), survival was significantly worse for the youngest group with P = 0.03. Age impacted other variables if patients were stratified by the mentioned age groups. Patients with positive receptors were stratified by age and the Kaplan Meier analysis showed that the youngest group is at a disadvantage P = 0.024. Among patients with positive LNs, those who are younger than 35 have worse survival, P = 0.021.

**Figure 2:**
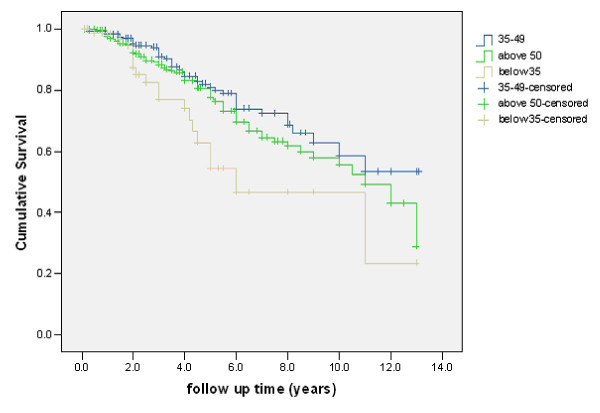
Kaplan-Meier survival analysis of patients with positive axillary lymph nodes and effects on survival stratified by age groups below 35 years, between 35-50 years, and above 50 years. Patients with positive axillary lymph nodes and young age have worst survival. (P=0.021).

Effects of age according to Lymph node status: We looked at the effects of young age in patients with positive lymph nodes versus negative lymph nodes, and patients with positive hormonal receptors versus negative hormonal receptors in the three different age groups below 35 years (group 1), between 35–50 (group 2) and above 50 (group 3). For patients with positive lymph nodes, age was a significant prognostic factor (Figure 2).

**Figure 3:**
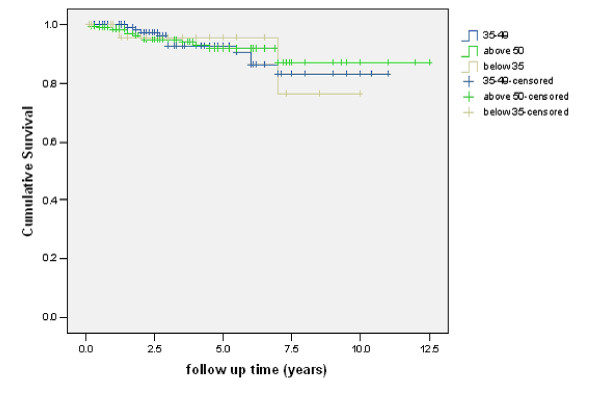
Kaplan-Meier survival analysis of patients with negative axillary lymph nodes and effects on survival stratified by age groups below 35 years, between 35-50 years, and above 50 years. Age had no effect on survival in groups with negative axillary lymph nodes (P=0.960).

For groups with negative LN, there was no impact on survival. The numbers of patients in the Kaplan Meier graph were as follows: Total number was 426 patients, 32 patients in group below 35 years, 158 patients in group 35–50 years, and 236 in group >50 years; and the number of events were 2, 11, and 14 in each of the age groups respectively. The survival curves for each of those subgroups did not differ significantly (Figure 3)

**Figure 4:**
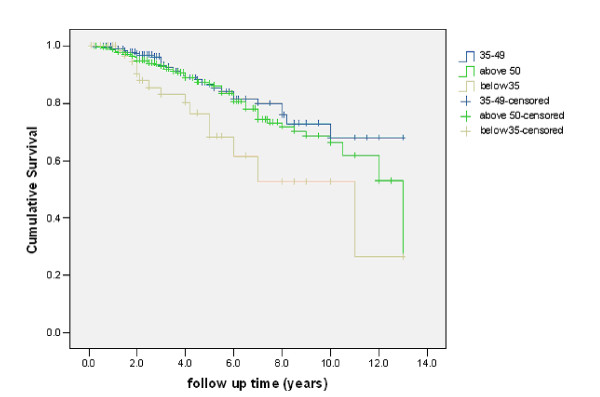
Kaplan-Meier survival analysis of patients with positive hormonal receptors and effects on survival stratified by age groups below 35 years, between 35-50 years, and above 50 years. Survival is worst in younger-aged patients with positive hormonal receptors (P=0.024).

**Figure 5:**
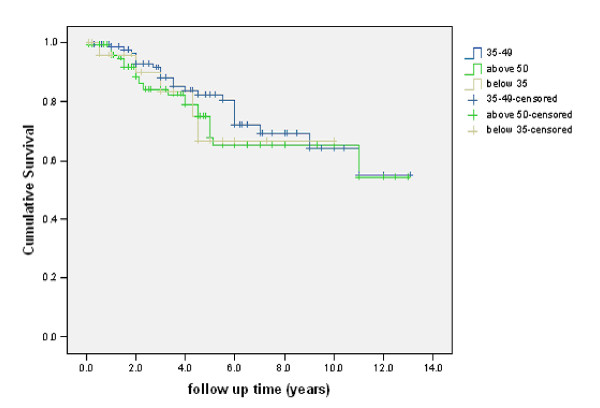
Kaplan-Meier survival analysis of patients with negative hormonal receptors and effects on survival stratified by age groups below 35 years, between 35-50 years, and above 50 years. Age had no effect on survival in groups of patients with negative hormonal receptors (P: 0.481).

Effects of age according to hormonal receptors status: Analysis of the three different age groups for patients with positive hormonal receptors showed the following numbers: 72 patients in group 1, 320 patients in group 2 and 481 patients in group 3. The total was 873 patients. We plotted survival curves and age was a significant prognostic factor (Figure 4). Analysis the three different age groups for patients with negative hormonal receptors showed the following numbers of patients in each age category in the Kaplan Meier: Total number of patients was 315 patients, below 35 years: 29 patients, between 35–50 years: 154 patients, and above 50 years there were 132 patients. We plotted survival graphs and there were no statistically significant difference in subgroups of patients with negative hormonal receptors (Fig 5).

## Discussion

Young age at diagnosis of breast cancer has been reported in many studies from the US and Europe to be an independent predictor of worse survival[4,6-9,11]. The risk factor profile of young patients is worse than their older counterparts. Young patients tend to have larger tumor sizes, more positive lymph nodes, more negative hormone receptors, higher tumor grades than their older counterparts[9,10,17-19]. The issue remains controversial and not all studies reported age as an adverse prognostic factor[10-13,17,18]. Few reports from developing countries such as from Saudi Arabia found that young age did not have an adverse effect on survival of breast cancer patients[12]. Chia et al, in a retrospective study of breast cancer patients, from Singapore, demonstrated that young females with breast cancer had a better survival than older females[13]. The recently reported population-based study from Switzerland showed no effect of young age on survival but authors had only 3% of their patients below age 35, 26% 35–50 and 71% above 50. This small number of very young patients may have affected statistical power of study. Authors concluded that when standard care is offered to young patients, their prognosis is not worse than their older counterparts[11].

Our results show that young age is a bad prognostic factor in women with breast cancer in Lebanon. The distribution of the various prognostic factors, except estrogen receptor positivity, and grade did not differ significantly in the three age groups in our patient population. A higher than expected percentage of receptor positivity was noted; this correlates with recent reports from other neighbouring countries and the USA[20,21]. Recent Surveillance Epidemiology and End Results (SEER) data indicated a 20% increase in the incidence of ER positive tumors between 1992 and 1998[20]. Similar increased percentage of higher estrogen receptor positivity in breast cancer was reported as well from Israel[6] and Kuwait[21]. Although our patients had an increased rate of positive receptors and received more aggressive chemotherapy than older patients, they still had a worse survival.

Although group 3 of older patients had significantly more hypertension (HTN), diabetes (DM) and cardiac diseases (CAD) (P < 0.001), no effect of co-morbid illnesses (CAD, DM, and HTN) were detected in the three age groups.

Velanovich et al [22] reported that older patients with breast cancer are usually undertreated because of co-morbid conditions (40.9%), or for refusal of treatment (31.8%), or favourable tumor pathology (13.8%) or unexplainable causes (13.6%). In our population, 53.1% of patients over 50 years of age received chemotherapy, as compared to 76.5% and 72.8% in the younger groups 1 and 2 respectively.

Overall data shows that young age is a significant prognostic factor for survival of patients with primary breast cancer. We looked at subgroups of patients with negative vs positive Lymph nodes and patients with negative vs. positive hormonal receptors and had different results. Young age had a significant impact on survival in patients with positive lymph nodes but not in patients with negative lymph nodes. Young age had a significant impact on survival in patients with positive receptors but not negative receptors. Grade and vascular invasion were significantly worse in group 2 (between 35–50 years) with positive LN vs. negative LN, and in group 3 (>50 years) there were significantly more vascular invasion in node positive versus node negative patients. The analysis of subgroups, in efforts to explain the differences, remains unresolved and requires further investigation.

## Conclusion

In conclusion, we report that young age is an independent adverse prognostic factor in younger patients with breast cancer. Younger patients had a percentage of positive hormone receptors that is higher than expected, but similar to recent trends reported in the literature. Younger patients received more anthracycline-based adjuvant chemotherapy. Survival in young patients was worse inspite the fact that they also received adjuvant hormonal therapy with tamoxifen. Young age at presentation remained a bad prognostic factor in patients with breast cancer. This negative impact on survival was seen in patients with positive lymph nodes and those with positive hormonal receptors.

## Abbreviations

AUBMC: American University of Beirut Medical Center

OCP: Oral Contraceptive Pill

HRT: Hormone Replacement Therapy

LN: Lymph Node

ER: Estrogen Receptor

PR: Progesterone Receptor

SEER: Surveillance Epidemiology and End Results

HTN: Hypertension

DM: Diabetes Mellitus

CAD: Coronary Artery Disease

## Competing interests

The author(s) declare that they have no competing interests.

## Authors' contributions

NSES contributed to the database, analysis, writing and editing of the manuscript. MS contributed to database, analysis, writing and editing of the manuscript. MKK contributed to data collection, writing and editing of the manuscript. MC contributed to data collection, statistical analysis, writing, and tables and graphs and editing of the manuscript. ZKS contributed to database and to the editing of the manuscript. FBG contributed to database, and editing of the manuscript. AIS contributed to database, analysis, writing and editing of the manuscript. All authors read and approved the final manuscript.

## Pre-publication history

The pre-publication history for this paper can be accessed here:



## Figures and Tables

**Table 1:** Clinical and pathological characteristics, hormonal receptor status, stages, and adjuvant chemotherapy of all patients grouped as <35 years (group 1), between 35–50 years (group 2) and >50 years (group 3).

**Variable**	**Group1 = <35**	**Group2 = 35–49**	**Group3 = >50**	**Total**	**P value**
**Tumor size<2 cm**	16 (15.7%)	95 (20.0%)	115 (18.8%)	1191	0.877
**Tumor 2–5 cm**	70 (68.6%)	315 (66.1%)	407 (66.4%)		
**> 5 cm**	16 (15.7%)	66 (13.9%)	91 (14.8%)		
					
**Lymph Node Positive**	70 (68.6%)	317 (66.7%)	378 (61.6 %)	1191	0.131
					
**Positive hormone Receptors (estrogen and/or progesterone)**	73 (71.6%)	321 (67.6%)	482 (78.5%)	876/1191	<0.001
					
**Grade I**	5 (4.9%)	49 (10.3%)	79 (12.9%)	1191	0.019
**Grade II**	47 (46.1%)	226 (47.6%)	315 (51.3%)		
**Grade III**	50 (49%)	200 (42.1%)	220 (35.8%)		
					
**Stage I**	12 (11.2%)	74 (14.8%)	96 (14.6%)	182 (14.4%)	0.619
**Stage II**	63 (58.9%)	305 (61.1%)	389 (59.1%)	757 (59.9%)	
**Stage III**	27 (25.2%)	96 (19.2%)	129 (19.7%)	252 (20.0%)	
Stage IV**	5 (4.7%)	24 (4.8%)	44 (6.6%)	72 (5.7%)	
					
**Adjuvant Chemotherapy**	78 (76.5%)	346 (72.8%)	326 (53.1%)	750/1191	<0.001
					
Adjuvant Anthracycline*	42 (56.0%)	228 (68.3%)	163 (50.6%)	443/731	<0.001
					
**Development of metastasis**	33 (32.4%)	109 (22.9%)	140 (22.8%)	282/1191	0.098

**Table 2:** Effects of various prognostic factors on survival: Cox Regression analysis of effects of tumor grade, positive hormonal receptors, tumor size and age on survival for non-metastatic patients. Patients with grade III had a significant higher risk of death (p 0.039) as compared to the reference grade I. Hormone receptor negativity conveys a significantly higher risk for death (p value 0.003). Larger tumor sizes and lymph node positivity correlated with significantly higher risks for death. Patients below 35 have the highest risk of death as compared to patients age 35–50 and those above 50 years. Other variables including adjuvant chemoradiation and hormonal therapy were not found to be significant.

**Variable**	**P value**	**Hazard Ratio**	**95% CI for hazard ratio**
Grade I	0.097	1.000	
Grade II	0.113	1.813	0.87 – 3.78
Grade III	0.039	2.161	1.04 – 4.49
Negative hormonal receptors	0.003	1.654	1.18 – 2.32
Tumor size < 2 cm	0.002	1.000	
Tumor size 2 cm – 5 cm	0.020	2.003	1.12 – 3.59
Tumor size >5 cm	0.001	3.099	1.63 – 5.89
Presence of positive Lymph Nodes	<0.0001	2.370	1.54 – 3.64
Age less than 35	0.015	1.000	
Age between 35–49	0.005	0.476	0.28 – 0.80
Age 50 and above	0.086	0.651	0.40 – 1.06

## References

[B1] American Cancer Society (2004). Cancer Facts and Figures 2004.

[B2] Adib SM, Mufarrij AA, Shamseddine AI, Kahwaji SG, Issa P, El-Saghir NS (1998). Cancer in Lebanon: An Epidemiological Review of the American University of Beirut Medical Center Tumor Registry(1983-1994). Annals of Epidemiology.

[B3] Shamseddine A, Sibai AM, Gehchan N, Rahal B, El-Saghir N, Ghosn M, Aftimos G, Chamsuddine N, M. S (2004). Cancer Incidence in Postwar Lebanon:Findings from the First National Population -based Registry, 1998. Ann Epidemiol.

[B4] Rodrý´guez-Cuevas S, Macý´as CG, Franceschi D, Labastida S (2001). Breast Carcinoma Presents a Decade Earlier in Mexican Women than in Women in the United States or European Countries. Cancer.

[B5] Ezzat AA, Ibrahim EM, Raja MA, Al-Sobhi S, Rostom A, Stuart RK (1999). Locally advanced breast cancer in Saudi Arabia: high frequency of stage III in a young population. Med Oncol.

[B6] Nissan A, Ram M, Spira MD, Hamburger T, Badrriyah M, Prus D, Cohen T, Hubert A, Freund HR, Peretz T (2004). Clinical profile of breast cancer in Arab and Jewish Women in the Jerusalem Area. Am J of Surgery.

[B7] El-Saghir NS, Shamseddine AI, Geara F, Bikhazi K, Rahal B, Salem ZM, Taher A, Tawil A, El Khatib Z, Abbas J, Hourani M, Seoud M (2002). Age Distribution of Breast Cancer in Lebanon: Increased Percentages and Age Adjusted Incidence Rates of Younger-Aged Groups at Presentation. J Med Liban.

[B8] Ries LAG, Eisner MP, Kosary CL, Hankey BF, Miller BA, Clegg L, Mariotto A, Feuer EJ, Edwards BK ( 2004). SEER Cancer Statistics Review, 1975-2001, National Cancer Institute. Bethesda, MD, http://seer.cancer.gov/csr/1975_2001/.

[B9] Swanson GM, Lin CS (1994). Survival Patterns among Younger Women with Breast Cancer: the Effects of Age, Race, Stage and Treatment. Monogr Natl Cancer Inst.

[B10] de la Rochefordiere A, Asselain B, Campana F, Scholl SM, Fenton J, Vilcoq JR, Durand JC, Pouillart P, Magdelenat H, Fourquet A (1993). Age as a Prognostic factor in Premenopausal Breast Carcinoma. Lancet.

[B11] Rapiti E, Fioretta G, Verkooijen HM, Vlastos G, Schafer P, Sappino AP, Kurtz J, Neyroud-Caspar I, Bouchardy C (2005). Survival of young and older breast cancer patients in Geneva from 1990 to 2001. Eur J Cancer.

[B12] al-Idrissi HY, Ibrahim EM, Kurashi NY, Sowayan SA (1992). Breast Cancer in a Low Risk Population.The Influence of Age and Menstrual Status on Disease Pattern and Survival in Saudi Arabia. Int J Cancer.

[B13] Chia KS, Du WB, Sankaranarayanan R, Sankila R, Wang H, Lee J, Seow A, Lee HP (2004). Do younger female breast cancer patients have a poorer prognosis? Results from a population-based survival analysis. Int J Cancer.

[B14] Arriagada R, Le MG, Guinebretiere JM, Dunant A, Rochard F, T. T (2003). Late local recurrences in a randomised trial comparing conservative treatment with total mastectomy in early breast cancer patients.. Ann Oncol.

[B15] Vrieling C, Collette L, Fourquet A, Hoogenraad WJ, Horiot JH, Jager JJ, Pierart M, Poortmans PM, Struikmans H, Maat B, Van Limbergen E, Bartelink H (2000). The influence of patient, tumor and treatment factors on the cosmetic results after breast-conserving therapy in the EORTC 'boost vs. no boost' trial. EORTC Radiotherapy and Breast Cancer Cooperative Groups.. Radiother Oncol.

[B16] van der Hage JA, Putter H, Bonnema J, Bartelink H, Therasse P, van de Velde CJEORTCBCG (2003). Impact of locoregional treatment on the early-stage breast cancer patients: a retrospective analysis.. Eur J Cancer.

[B17] Kroman N, Jensen MB, Wohlfahrt J, Mouridsen HT, Andersen PK, Melbye M (2000). Factors influencing the effect of age on prognosis in breast cancer:population based study. BMJ.

[B18] Shannon C, Smith IE (2003). Breast cancer in adolescents and young women. Eur J Cancer.

[B19] Albain KS, Allred C, Clark GM (1994). Breast Cancer Outcome and Predictors of Outcome: Are There Age Differentials. J Natl Cancer Inst Monogr.

[B20] Li CI, Daling JR, Malone KE (2003). Incidence of invasive breast cancer by hormone receptor status from 1992 to 1998. J Clin Oncol.

[B21] Paszko Z, Omar YT, Nasralla MY, Jazzaf H, Bouzubar N, Temmim L, Padzik H (1993). Estrogen and progesterone receptor status in breast cancer in Kuwait female population. Neoplasma.

[B22] Velanovich V, Gabel M, Walker EM, Doyle TJ, O'Bryan RM, Szymanski W, Ferrara JJ, Lewis FRJ (2002). Causes for the undertreatment of elderly breast cancer patients: tailoring treatments to individual patients. J Am Coll Surg.

